# TDP-43 Accumulation Within Intramuscular Nerve Bundles of Patients With Amyotrophic Lateral Sclerosis

**DOI:** 10.1001/jamaneurol.2022.1113

**Published:** 2022-05-23

**Authors:** Takashi Kurashige, Hiroyuki Morino, Tomomi Murao, Yuishin Izumi, Tomohito Sugiura, Kazuya Kuraoka, Hideshi Kawakami, Tsuyoshi Torii, Hirofumi Maruyama

**Affiliations:** 1 Department of Neurology, National Hospital Organization Kure Medical Center and Chugoku Cancer Center, Kure, Japan; 2 Department of Clinical Neuroscience and Therapeutics, Graduate School of Biomedical and Health Sciences, Hiroshima University, Hiroshima, Japan; 3 Department of Clinical Neuroscience, Institute of Biomedical Sciences, Tokushima University Graduate School, Tokushima, Japan; 4 Department of Diagnostic Pathology, National Hospital Organization Kure Medical Center and Chugoku Cancer Center, Kure, Japan; 5 Department of Epidemiology, Research Institute for Radiation Biology and Medicine, Hiroshima University, Hiroshima, Japan

## Abstract

**Importance:**

Degeneration of neuromuscular junctions and axons is considered an important aspect of the pathomechanism of amyotrophic lateral sclerosis (ALS). However, a mechanism including the role of transactive response DNA-binding protein 43 (TDP-43) in axons has not been pathologically clarified.

**Objective:**

To identify and characterize the histopathology of peripheral axons in the skeletal muscle of patients with ALS.

**Design, Setting, and Participants:**

This study comprised 2 parts: a postmortem case-control study and a retrospective population-based cohort study with a minimum of 1 year of follow-up. Patients in the cohort study were enrolled from January 1, 2004, to September 30, 2019. The postmortem study included patients with sporadic ALS (SALS) with TDP-43 pathology and control patients with non-ALS disease. The cohort study enrolled patients without a family history of ALS or other neuromuscular disease and those not diagnosed with a muscle disease at biopsy. Patients were excluded if their clinical records were not screened after biopsy, if they were diagnosed with a muscular disease, and if they were harboring known causative genes of ALS. Data were collected between September 2019 and June 2021 and analyzed in June 2021.

**Exposures:**

Muscle biopsy or postmortem muscle tissue examination.

**Main Outcomes and Measures:**

Clinical information and muscle pathological characteristics.

**Results:**

A total of 10 patients with autopsy-confirmed SALS (mean [SD] age at death, 76.1 [8.5] years; 8 men [80%]) exhibited axonal phosphorylated TDP-43 (pTDP-43)–positive accumulations in intramuscular nerve bundles; the 12 control patients without ALS did not. Among the 114 patients in the cohort study (mean [SD] age, 62.3 [16.1] years; 76 men [67%]), 71 patients (62.3%) exhibited intramuscular nerve bundles; 43 (37.7%) did not. Among those who exhibited pTDP-43–positive intramuscular nerve bundles, 33 patients (22 men [66.7%]; mean [SD] age, 65.2 [15.6] years) were later diagnosed with ALS. The other 38 patients (26 men [68.4%]; mean [SD] age, 59.3 [18.0] years) showed no pTDP-43–positive bundles and did not develop ALS. Among those without evident nerve bundles (28 men [65.1%]; mean [SD] age, 61.3 [15.3] years), 3 were later diagnosed with ALS. Among patients with ALS in the biopsy cohort, 9 with pTDP-43–positive bundles showed only lower motor neuron symptoms at biopsy.

**Conclusions and Relevance:**

Results of this dual case-control and retrospective cohort study suggest that axonal pTDP-43 accumulations may be characteristic for patients with ALS. As such findings precede clinical fulfillment of the Gold Coast criteria, TDP-43 in nerve bundles may be a novel diagnostic biomarker for ALS.

## Key Points

**Question:**

Does a characteristic pathological hallmark exist in the muscle tissues of those with amyotrophic lateral sclerosis (ALS)?

**Findings:**

In this dual case-control study of 10 autopsied patients and cohort study of 114 consecutive patients, those with sporadic ALS (SALS) were confirmed to have phosphorylated transactive response DNA-binding protein 43 (pTDP-43)–positive axons in intramuscular nerve bundles. In 114 patients without a family history of ALS or other neuromuscular diseases for whom muscle biopsies were performed, all patients with axonal pTDP-43–positive nerve bundles were diagnosed with SALS after biopsy.

**Meaning:**

Results of this study suggest that axonal pTDP-43 accumulation in intramuscular nerve bundles may be characteristic for patients with ALS.

## Teaser

This dual case-control and cohort study assesses for a characteristic pathological hallmark in the muscle tissues of patients with amyotrophic lateral sclerosis.

## Introduction

Amyotrophic lateral sclerosis (ALS) is a clinical diagnosis based on a history of progressive motor dysfunction that demonstrates a combination of upper motor neuron (UMN) and lower motor neuron (LMN) signs.^1^ The vast majority of ALS cases are pathologically characterized by the deposition of abnormal ubiquitinated inclusions immunoreactive to transactive response DNA-binding protein 43 (TDP-43).^1^ ALS with TDP-43 pathology features axonal phosphorylated TDP-43 (pTDP-43) aggregates predominantly located in the facial and hypoglossal nuclei and anterior horn cells. These aggregates may influence neuron function; moreover, presynaptic aggregates induce loss of TDP-43 in the nuclei of postsynaptic neurons.^6^ Axonal degeneration, loss of peripheral axons, and destruction of nerve terminals (including neuromuscular junctions) are regarded as the basis of LMN dysfunction; such destruction begins from the early stage of the disease pathogenic cascade.^7^ However, histopathology relating to neuromuscular junctions and spinal anterior horn neurons has not focused on the degeneration of neuromuscular junctions; rather, axons have been considered a critical element of the ALS pathomechanism.^7^ Based on a mouse model, TDP-43 has been reported to play roles in the axonal transport of ribonucleoprotein and ribosomal protein messenger RNA and in maintaining the functionality of axonal ribosomes and mitochondria,^12^ which are required for local protein synthesis in response to axon stimulation and stress.^13^ Although pathological changes of the central nervous system are only accessible at postmortem examination, myopathological findings can be assessed premortem; consequently, it is very important to describe findings specific for ALS, which will enable us to understand the pathomechanism underlying LMN dysfunction in this disease. Previous studies did not focus on intramuscular axons,^16^ but Altmann et al^15^ recently reported the observation of dotlike TDP-43 accumulations in the intramuscular axons of 3 patients diagnosed with clinically definite or probable ALS according to El Escorial criteria. The specificity of this finding has not been confirmed because muscle specimens of patients with autopsy-confirmed ALS and patients with ALS mimics were not examined.^15^

Here, we describe the pathological changes associated with TDP-43 accumulation in the intramuscular nerve bundles in a postmortem study and a muscle biopsy cohort of patients so as to characterize myopathological findings that may be characteristic for ALS.

## Methods

### Ethical Considerations

The study was approved by the institutional review boards of Hiroshima University and the National Hospital Organization Kure Medical Center and Chugoku Cancer Center and adhered to the Standards for Reporting of Diagnostic Accuracy (STARD) reporting guidelines. All examinations were performed after having obtained written informed consent provided by all participants or their families for diagnostic purpose followed by research application.

### Study Design and Participants

Our investigations were performed using a 2-step approach. First, we examined muscle tissues in the postmortem case-control study patients with sporadic ALS (SALS) and patients without ALS at the National Hospital Organization Kure Medical Center and Chugoku Cancer Center. Patients with SALS had neither any family history of ALS or other neuromuscular diseases nor pathological variants in the known causative genes of ALS. These patients were confirmed to have the TDP-43 pathology by postmortem examination. Control skeletal muscle specimens were obtained from patients without ALS and without incidental TDP-43 pathology. Muscle tissues of the tongue, diaphragm, biceps brachii muscle, or rectus femoris muscle obtained from patients with autopsy-confirmed SALS and patients without ALS were examined as either formalin-fixed, paraffin-embedded sections or frozen sections fixed by liquid nitrogen–cooled isopentane.

In the second part of the study, we examined the muscle biopsy cohort of patients who underwent open muscle biopsy for diagnostic purposes at the National Hospital Organization Kure Medical Center and Chugoku Cancer Center or Hiroshima University Hospital between January 1, 2004, and September 30, 2019. All muscle biopsy specimens were frozen in liquid nitrogen–cooled isopentane for histochemistry and immunohistochemistry. Pathological diagnosis was confirmed by routine histochemistry and immunohistochemical analysis. These patients were screened for various neuromuscular diseases whose clinical records from after the muscle biopsy were available for reference. We excluded any patients who were diagnosed as having hereditary or acquired muscle diseases. In addition, we excluded patients harboring pathogenic variants in causative genes of ALS. The remaining patients were not diagnosed as having muscle disease by biopsy or other examination and did not have any family history of ALS or other neuromuscular diseases; we analyzed the muscle biopsy specimens and clinical records of these remaining patients. The latest diagnoses of these patients were confirmed within a mean (SD) of 4.7 (4.8) months after muscle biopsy. Clinical symptoms and electrophysiological manifestations at muscle biopsy were categorized using the Gold Coast criteria.^16^

For analyzing both cohorts, 2 observers (T.K., T.M.) accessed myopathological findings individually, and if required, a joint assessment was scrutinized under a 2-headed microscope. These 2 observers were not informed of the diagnoses and clinical manifestations of the patients, which were collected by other coauthors.

### Immunohistochemistry

For each muscle specimen, 8-μm transverse sections were subjected to histochemistry and immunohistochemistry. These sections were immunostained using a Ventana BenchMark GX automated slide staining system (Ventana Medical Systems) with a mouse monoclonal pTDP-43 antibody (TIP-PTD-M01, pSer409/410, 1:3000 [CosmoBio]) and a rabbit polyclonal pTDP-43 antibody (22309-1-AP, pSer409/410, 1:2000 [Proteintech]) according to manufacturer instructions. Antibody dilutions were determined by referring to pTDP-43–immunopositivities in the spinal cords and motor cortexes of both patients with ALS and those without ALS. These antibodies were used not only for detecting accumulations but also for excluding nonspecific findings. If the initial section did not contain intramuscular nerve bundles, we made at least 5 prospective step sections of different levels at 50-μm intervals so as to perform a thorough evaluation for intramuscular nerve bundles.

### Immunofluorescence

Immunofluorescence detection was performed on 8-μm transverse sections at 50-μm intervals. After washing in phosphate-buffered saline, the sections were incubated with primary mouse and rabbit antibodies overnight at 4 °C and afterward directly visualized using an antimouse secondary antibody conjugated with Alexa Fluor 568 (A11004, 1:1000 [Life Technologies]) and an antirabbit secondary antibody conjugated with Alexa Fluor 488 (A11034, 1:1000 [Life Technologies]). The primary antibodies consisted of mouse monoclonal antibodies against pTDP43 antibody (pSer409/410, 1:3000 [CosmoBio]), p62 (ab56416, 1:1000 [Abcam]) and ubiquitin (MAB1510, 1:2000 [Millipore]); rabbit polyclonal antibodies against the fused in sarcoma (FUS) protein (A300-302A, 1:100 [Bethyl Laboratories]) and TDP-43 (12892-1-AP, 1:3000 [Proteintech]); and goat polyclonal antibody against 160 kD neurofilament (ab195658, 1:100 [Abcam]). Sections were photographed using a BIOREVO BZ-9000 fluorescence microscope (Keyence).

### Statistical Analysis

All values are expressed as mean (SD) unless stated otherwise. Differences among means were analyzed with a *t* test, the Kruskal-Wallis test, the Mann-Whitney *U* test, the χ^2^ test, Pearson correlation coefficient, or 1-way analysis of variance using Prism 8 software (GraphPad Software). All *P* values except for the Kruskal-Wallis test were 2-sided, and a *P* value < .05 was considered significant. The data for this study were analyzed in June 2021.

## Results

**Figure 1.**
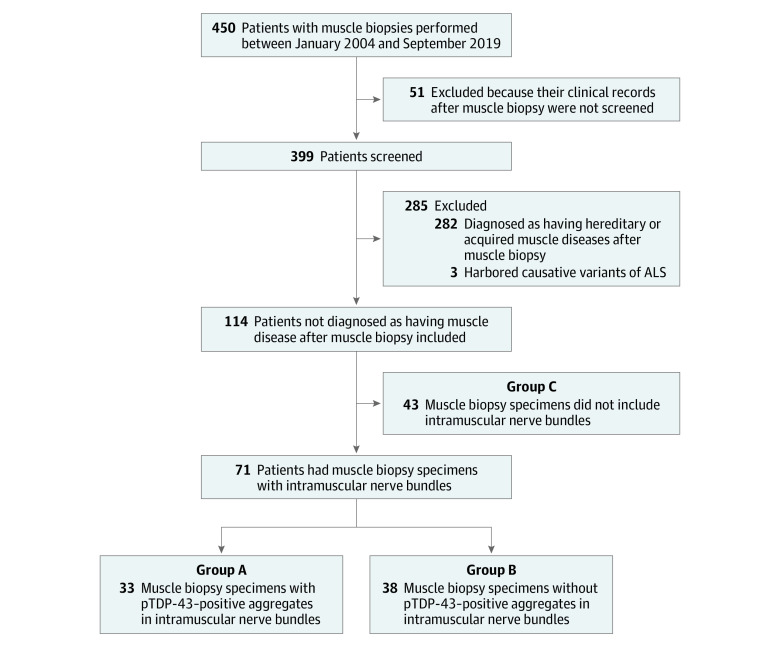
Study Protocol in the Muscle Biopsy Cohort of Patients Whose Muscle Biopsies Were Performed Between January 2004 and September 2019 ALS indicates amyotrophic lateral sclerosis; pTDP-43, phosphorylated transactive response DNA-binding protein 43.

**Figure 2.**
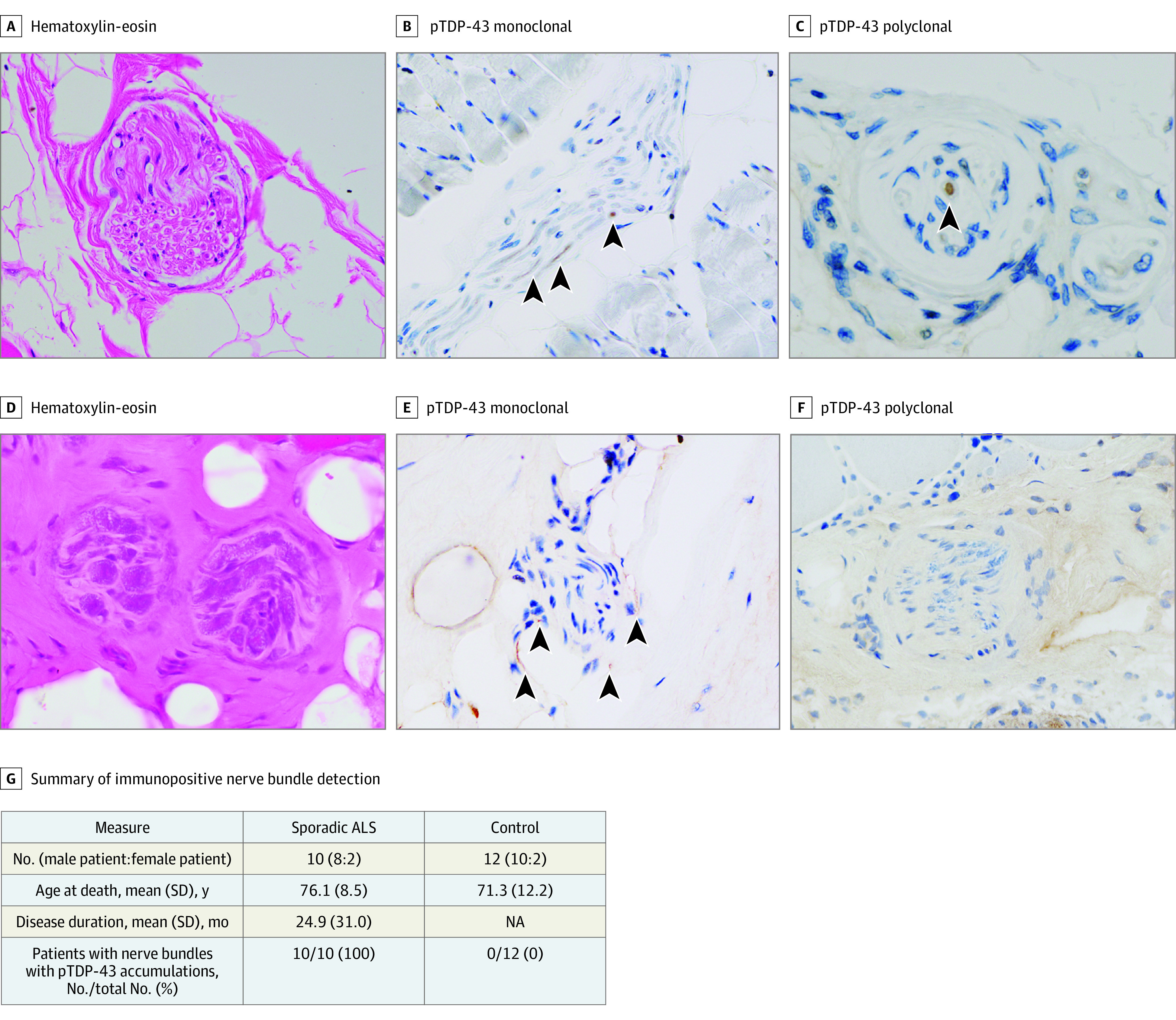
Axonal Phosphorylated Transactive Response DNA-Binding Protein 43 (pTDP-43)–Positive Accumulations in Intramuscular Nerve Bundles of Skeletal Muscle in the Postmortem Case-Control Study A, D, Hematoxylin-eosin staining in axons of intramuscular nerve bundles showed no differences between patients with amyotrophic lateral sclerosis (ALS) and those without ALS except for the density of intramuscular nerves (original magnification: A, ×40; D, ×60). B, Immunohistochemical analysis with mouse monoclonal pTDP-43–positive accumulations in intramuscular nerve bundles (arrowheads) of patients with spontaneous ALS (SALS) (original magnification, ×40). E, Immunostaining with mouse monoclonal antibody against pTDP-43 in patients with non-ALS diseases did not reveal any abnormalities in intramuscular nerve bundles (original magnification, ×40). C, Immunohistochemical analysis with rabbit polyclonal pTDP-43 antibody also revealed pTDP-43–positive accumulations in intramuscular nerve bundles (arrowheads) of patients with SALS (original magnification, ×60). F, Immunostaining with rabbit polyclonal antibody against pTDP-43 in patients with non-ALS diseases did not reveal any abnormalities in intramuscular nerve bundles (original magnification, ×40). G, Summary of immunopositive nerve bundle detection in patients with autopsy-confirmed ALS. NA indicates not applicable.

A total of 10 patients with autopsy-confirmed SALS (mean [SD] age at death, 76.1 [8.5] years; 8 men [80%]; 2 women [20%]) exhibited pTDP-43–positive accumulations in intramuscular nerve bundles; the 12 control patients without ALS did not. The study protocol for the muscle biopsy cohort is illustrated in Figure 1, and the clinical and pathological characteristics of autopsy-confirmed patients are described in Figure 2G and the eTable in the Supplement.

Although hematoxylin-eosin staining showed no differences between patients with SALS and the control patients without ALS regarding the axons of intramuscular nerve bundles, except in terms of the density of intramuscular nerves (Figure 2A and 2D), immunohistochemical analysis revealed pTDP-43–positive accumulations in approximately one-half of the intramuscular nerve bundles of patients with SALS (Figure 2B and 2C). Control patients without ALS did not exhibit any pTDP-43–positive nerve bundles (Figure 2E and 2F).

In the intramuscular nerve bundles of patients with SALS, accumulations positive for pTDP-43 or TDP-43 colocalized with the axons stained by antineurofilament antibody (eFigure 1A and eFigure 2 in the Supplement). In addition, pTDP-43 accumulations were scattered in perimysial axons present outside the nerve bundles, consistent with a previous report^15^ (eFigure 1B in the Supplement). In contrast, FUS-, p62-, and ubiquitin-positive accumulations were not observed in the nerve bundles of patients with SALS (eFigures 3, 4, and 5 in the Supplement). Control patients likewise showed no accumulations in intramuscular nerve bundles that stained for pTDP-43, TDP-43, FUS, p62, or ubiquitin (eFigures 1, 2, 3, 4, and 5 in the Supplement).

Among patients with SALS, statistical analysis did not reveal any significant difference in the percentage of nerve bundles with pTDP-43 accumulations across 4 types of muscle (ie, tongue, diaphragm, biceps brachii, and iliopsoas muscles) (eFigure 6 in the Supplement).

### pTDP-43 in Muscle Biopsy Specimens

**Figure 3.**
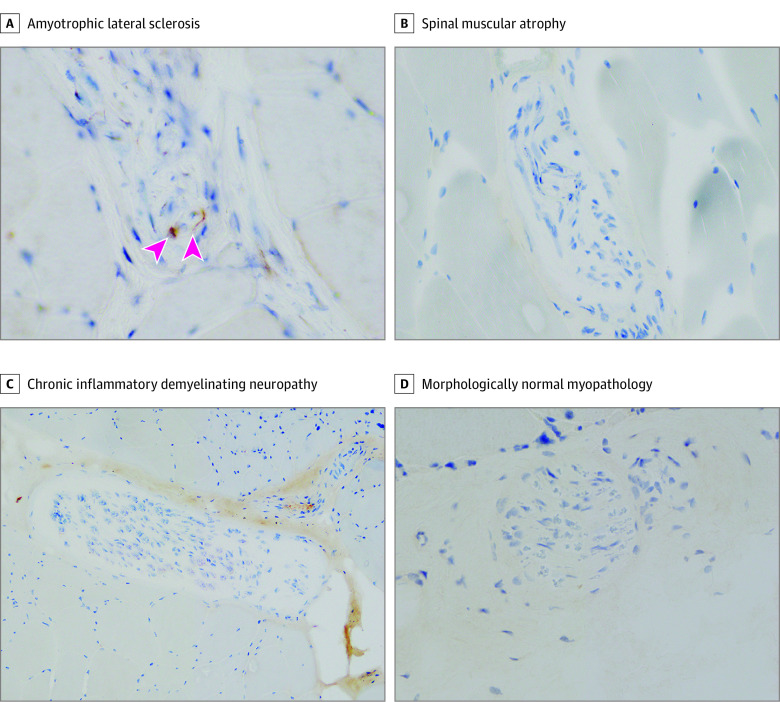
Axonal Phosphorylated Transactive Response DNA-Binding Protein 43 (pTDP-43)–Positive Accumulations Appeared in Intramuscular Nerve Bundles of Patients Who Ultimately Developed Amyotrophic Lateral Sclerosis (ALS) in the Muscle Biopsy Cohort A, pTDP-43 accumulations (arrowheads) were evident in intramuscular nerve bundles from patients ultimately diagnosed with ALS (original magnification, ×40). B-D, pTDP-43–positive accumulations were not observed in intramuscular nerve bundles of patients with spinal muscular atrophy (B), chronic inflammatory demyelinating neuropathy (C), or morphologically normal myopathology (D) (original magnification: B-C, ×20; D, ×40).

In the second part of the study, we examined the muscle biopsy cohort of 450 consecutive patients who underwent open muscle biopsy. Of these patients, we screened 399 with various neuromuscular diseases whose clinical records from after the muscle biopsy were available for reference. We excluded 282 patients because they were diagnosed as having hereditary or acquired muscle diseases. In addition, we excluded 3 patients harboring pathogenic variants in causative genes of ALS. This left 114 patients who were not diagnosed as having muscle disease by biopsy or other examination and did not have any family history of ALS or other neuromuscular diseases as per the study protocol for muscle biopsy specimens (Figure 1). The clinical and pathological characteristics of these patients are described in Table 1. Among the 114 patients in the muscle biopsy cohort (mean [SD] age, 62.3 [16.1] years; 76 men [67%]; 38 women [33%]), 71 patients (62.3%) exhibited intramuscular nerve bundles; 43 (37.7%) did not. Among those who exhibited pTDP-43–positive intramuscular nerve bundles, 33 patients (group A; 22 men [66.7%]; 11 women [33.3%]; mean [SD] age, 65.2 [15.6] years) featured axonal pTDP-43–positive accumulations in bundles (Figure 3A; eFigure 7 in the Supplement) and were later diagnosed with ALS. The other 38 patients (group B; 26 men [68.4%]; 12 women [31.6%]; mean [SD] age, 59.3 [18.0] years) showed no pTDP-43–positive bundles and did not develop ALS (Figure 3B-D; eFigure 7 in the Supplement). The remaining 43 patients without evident nerve bundles (group C; 28 men [65.1%]; 15 women [34.9%]; mean [SD] age, 61.3 [15.3] years) featured no intramuscular nerve bundles in either initial or step sections of their muscle biopsy specimens; 3 were later diagnosed with ALS. Among patients with ALS in the biopsy cohort, 9 with pTDP-43–positive bundles showed only LMN symptoms at biopsy. For 37 (52.1%) of those patients, intramuscular nerve bundles were not evident in the initial sections; subsequent step sections were required to observe them. 

**Table 1.** Demographic, Clinical, and Pathological Characteristics of the Muscle Biopsy Cohort

Characteristic	No./total No. (%)		
	Group A: patients with pTDP-43–positive nerve bundles (n = 33)	Group B: patients with pTDP-43–negative nerve bundles (n = 38)	Group C: patients without nerve bundles (n = 43)
Sex			
Men	22/33 (66.7)	26/38 (68.4)	28/43 (65.1)
Women	11/33 (33.3)	12/38 (31.6)	15/43 (34.9)
Age at biopsy, mean (SD), y	65.2 (15.6)	59.3 (18.0)	61.3 (15.3)
Disease duration until muscle biopsy, mean (SD), mo	20.4 (21.1)	23.2 (31.7)	21.1 (33.7)
Clinical diagnosis before biopsy			
ALS	7/33 (21.2)	0/38	2/43 (4.8)
LMN dysfunction exists in			
1 Region (not fulfilled with criteria)	1/33 (3.0)	0/38	0/43
≥2 Regions	0/33	0/38	0/43
UMN and LMN dysfunctions exist	6/33 (18.2)	0/38	2/43 (4.8)
SMA	0/33	0/38	1/43 (2.4)
CIDP/MMN	0/33	10/38 (26.3)	8/43 (19.0)
Vasculitic neuropathy	0/33	9/38 (23.7)	12/43 (26.2)
Hereditary neuropathy	2/33 (6.1)	4/38 (10.5)	2/43 (4.8)
Other neuropathies	4/33 (12.1)	12/38 (31.6)	8/43 (19.0)
Mitochondrial disorders	0/33	1/38 (2.6)	1/43 (2.4)
Muscle diseases	20/33 (60.6)	2/38 (5.3)	9/43 (21.4)
IBM	8/33 (24.2)	0/38	2/43 (4.8)
Other^a^	12/33 (36.4)	2/38 (5.3)	7/43 (16.3)
Clinical symptoms evaluated by Gold Coast criterion at biopsy			
Not fulfilled	3/33 (9.1)	16/38 (42.1)	25/43 (58.1)
LMN dysfunction exists in ≥2 regions	6/33 (18.2)	22/38 (57.9)	15/43 (34.9)
UMN and LMN dysfunctions exist	24/33 (36.4)	0/38	3/43 (7.0)
Diagnosis after muscle biopsy			
ALS^b^	33/33 (100)	0/38	3/43 (7.0)
SMA	0/33	2/38 (5.3)	2/43 (4.7)
CIDP/MMN	0/33	12/38 (31.6)	12/43 (27.9)
Vasculitic neuropathy	0/33	11/38 (28.9)	7/43 (16.3)
Hereditary neuropathy	0/33	6/38 (15.8)	1/43 (2.3)
Other neuropathies	0/33	7/38 (18.4)	18/43 (41.9)
Myopathological findings			
Grouped atrophy	30/33 (91.4)	25/38 (66.7)	29/43 (67.4)
Small	23/33 (68.6)	9/38 (25.6)	18/43 (41.9)
Large	7/33 (22.9)	16/38 (41.0)	11/43 (25.6)
Pyknotic nuclear clump	23/33 (68.6)	23/38 (61.5)	21/43 (48.8)
Fiber type grouping	26/33 (77.1)	25/38 (64.1)	30/43 (69.8)
Density of myelinated nerves in nerve bundle			
Preserved	13/33 (40.0)	22/38 (56.4)	
Decreased	20/33 (60.0)	16/38 (43.6)	
Not included	0/33	0/38	43/43 (100.0)
Necessity of step-cutting for the evaluation of nerve bundles	19/33 (57.6)	18/38 (48.7)	

Abbreviations: ALS, amyotrophic lateral sclerosis; CIDP, chronic inflammatory demyelinating polyneuropathy; IBM, inclusion body myositis; LMN, lower motor neuron; MMN, multifocal motor neuropathy; pTDP-43, phosphorylated transactive response DNA-binding protein 43; SMA, spinal muscular atrophy; UMN, upper motor neuron.

^a^ Other includes mitochondrial disorders, inflammatory myopathy, and congenital myopathy; muscular dystrophy was not included.

^b^ Clinical symptoms and electrophysiological manifestations at muscle biopsy were categorized using the Gold Coast criteria.

Genetic analysis was performed in all 33 patients (100%) of group A, 13 (34.2%) in group B, and 13 (30.2%) in group C. All tested patients were negative for pathological variants in known causative genes of ALS. There were no differences between the 3 groups in terms of sex, age at biopsy, or disease duration before muscle biopsy. Myopathological findings did not show any differences between 3 groups except for grouped atrophy.

### Axonal pTDP-43 in Intramuscular Nerve Bundles of Patients Later Diagnosed With ALS

In this cohort of 114 patients, only 9 were clinically suspected as having ALS before their muscle biopsy (Table 1). Using the most recent examination findings, we evaluated the clinical symptoms of included patients according to the Gold Coast criteria. Among the 33 patients in group A, 24 (72.7%) were classified as having UMN and LMN symptoms in at least 1 body region before their muscle biopsy, which fulfilled the Gold Coast criteria. The remaining 9 patients were without UMN symptoms, of which 6 fulfilled the Gold Coast criteria, and 3 showed LMN symptoms in only 1 region. Meanwhile, among the 38 patients in group B and the 43 in group C, only 3 patients had UMN and LMN symptoms at their muscle biopsy, and these were observed in at least 2 body regions.

All 33 patients in group A were diagnosed with ALS on the basis of their clinical and electrophysiological manifestations and investigations performed after their muscle biopsy that excluded other diseases (eFigure 8 in the Supplement). Within this group, 3 patients did not exhibit grouped atrophy in their muscle biopsy specimens. Meanwhile, the 38 patients in group B were all subsequently diagnosed as having other motor neuron diseases or neuropathies, including spinal muscular atrophy (Figure 3B; eFigure 7 in the Supplement), chronic inflammatory demyelinating polyneuropathy (Figure 3C; eFigure 7 in the Supplement), and neuropathies with morphologically normal muscles (Figure 3D; eFigure 7 in the Supplement). Of the 43 participants in group C, 3 were identified as patients with ALS (7.0%) for presenting with UMN and LMN symptoms in at least 2 regions as described previously. The remaining 40 patients (93.0%) were also diagnosed as having other motor neuron diseases or neuropathies (Table 1).

### Axonal pTDP-43 in the Intramuscular Nerve Bundles of Patients With ALS Not Fulfilling the Gold Coast Criteria

We analyzed the clinical and pathological characteristics of 36 patients with SALS who were diagnosed after muscle biopsy according to the clinical categories of the Gold Coast criteria (Table 2). Three patients (2 men [66.7%]; mean [SD] age, 64.0 [17.7] years) presented with LMN symptoms in only 1 region at biopsy, which did not fulfill the Gold Coast criteria. Six patients (3 men [50.0%]; mean [SD] age, 71.2 [10.1] years) showed LMN symptoms in 2 or more regions. The remaining 27 patients (20 men [74.1%]; mean [SD] age, 67.1 [13.6] years) had clinical and electrophysiological manifestations of both UMN and LMN dysfunctions in at least 1 region at biopsy. Patients with ALS exhibiting UMN and LMN symptoms at biopsy had shorter time intervals between muscle biopsy the time of diagnosis (mean [SD] time, 4.3 [3.6] months) than those with only LMN symptoms in 1 region (8.6 [3.1] months) or at least 2 regions (8.3 [2.6] months). Sex, age at biopsy, disease duration before muscle biopsy, and the clinical diagnosis suspected before biopsy were not associated with classification according to the Gold Coast criteria.

**Table 2.** Demographic, Clinical, Pathological Characteristics of 36 Patients With Spontaneous ALS in the Muscle Biopsy Cohort

Gold Coast criteria at biopsy	LMN dysfunction only		UMN and LMN dysfunction
	1 Region (not fulfilled)	≥2 Regions	
No.	3	6	27
Sex			
Men	2/3 (66.7)	3/6 (50.0)	20/27 (74.1)
Women	1/3 (33.3)	3/6 (50.0)	7/27 (25.9)
Age at biopsy, mean (SD), y	64.0 (17.7)	71.2 (10.1)	67.1 (13.6)
Disease duration, mean (SD), mo			
Until biopsy	19.0 (16.1)	9.5 (3.8)	22.1 (22.5)
Between biopsy to diagnosis	8.6 (3.1)	8.3 (2.6)	4.3 (3.6)
Clinical diagnosis before biopsy			
ALS^a^	0/3	1/6 (16.7)	8/27 (29.6)
Not fulfilled	0/3	1/6 (16.7)	0/27
Possible	0/3	0/6	1/27 (3.7)
Probable	0/3	0/6	7/27 (25.9)
Definite	0/3	0/6	0/27
CIDP/MMN	0/3	0/6	0/27
Hereditary neuropathy	0/3	0/6	2/27 (7.4)
Other neuropathies	1/3 (33.3)	2/6 (33.3)	1/27 (3.7)
Muscle diseases	2/3 (66.7)	3/6 (50.0)	16/27 (59.3)
IBM	0/3	0/6	9/27 (33.3)
Other^b^	2/3 (66.7)	3/6 (50.0)	7/27 (25.9)
EMG findings of biopsied muscles			
Myopathic	2/3 (66.7)	3/6 (50.0)	8/27 (29.6)
Chronic neuropathic	1/3 (33.3)	3/6 (50.0)	15/27 (55.6)
Neuropathic with active denervation	0/3	0/6	4/27 (14.8)
Patients with pTDP-43–positive nerve bundles	3/3 (100.0)	6/6 (100.0)	24/27 (88.9)
Positivity in nerve bundles, mean (SD), %	57.4 (17.5)	47.2 (23.9)	78.0 (24.9)
Necessity of step-cutting for the evaluation of nerve bundles	1/3 (33.3)	1/6 (16.7)	20/27 (74.1)
Step sections undetected bundles	0/3	0/6	3/27 (11.1)

Abbreviations: ALS, amyotrophic lateral sclerosis; CIDP, chronic inflammatory demyelinating polyneuropathy; IBM, inclusion body myositis; MMN, multifocal motor neuropathy; pTDP-43, phosphorylated transactive response DNA-binding protein 43; SMA, spinal muscular atrophy.

^a^ Clinical diagnosis of ALS was suspected according to the Awaji criteria.

^b^ Other includes mitochondrial disorders, inflammatory myopathy, and congenital myopathy; muscular dystrophy was not included.

Among patients with SALS, immunopositivity against pTDP-43 increased with Gold Coast categorization (eFigure 9 in the Supplement), although the 3 categories exhibited no statistical difference. The need for step sections when describing nerve bundles was also increased with Gold Coast category (Table 2). Among 27 participants with both UMN and LMN symptoms in at least 1 region at biopsy, we could not detect intramuscular nerve bundles in either the initial or additional sections of 3 patients (11.1%).

We additionally analyzed 71 patients in groups A and B with regard to their Gold Coast categories at biopsy (eFigure 9 in the Supplement). pTDP-43–positive intramuscular nerve bundles were observed not only in all patients with both UMN and LMN symptoms, but also in 19.1% (9 of 47 patients) of patients without UMN symptoms. Among participants without UMN symptoms, the proportion having pTDP-43–positive intramuscular nerve bundles did not differ between those with LMN symptoms in 2 or more regions and those having symptoms in only 1 region.

## Discussion

Results of this dual case-control and cohort study revealed axonal pTDP-43 accumulations in intramuscular nerve bundles among the muscle tissues of patients with autopsy-confirmed SALS. Such accumulations were not observed in patients without ALS at autopsy. Among our muscle biopsy cohort, axonal pTDP-43 accumulations appeared not only in specimens derived from patients with both UMN and LMN symptoms but also in approximately 20% of patients with only LMN symptoms. Among patients without UMN dysfunctions and with axonal pTDP-43 accumulations, 3 patients with pTDP-43–positive intramuscular nerve bundles showed LMN manifestations in only 1 region. Meanwhile, the difficulty of detecting nerve bundles increased significantly in patients with both UMN and LMN symptoms (Table 2).

The vast majority of SALS is characterized by deposition of abnormal ubiquitinated inclusions immunoreactive to TDP-43. These aggregates form neuronal cytoplasmic inclusions in the cell bodies of motor neurons, including skeinlike and round inclusions, neuronal intranuclear inclusions, and neuronal dystrophic neurites in axons.^1^ Dystrophic neurites are located predominantly in the axons of facial and hypoglossal nuclei and anterior horn cells and are considered to influence neuron function.^6^ The histopathology associated with spinal anterior horn neurons and neuromuscular junctions has not been adequately elucidated, although degeneration of neuromuscular junctions and axons is considered an important part of the ALS pathomechanism.^7^ Previous studies evaluating the muscle tissues of patients with autopsy-confirmed ALS also reported pTDP-43 accumulations in muscle fiber cytoplasm but did not focus on axons.^18^ However, Altman et al^15^ recently reported axonal dotlike accumulations of pTDP-43 and Ras guanosine triphosphate (GTP)ase-activating protein binding protein, a tunable protein of stress granules, in the intramuscular axons of muscle biopsy specimens from patients with SALS having both UMN and LMN manifestations in at least 2 regions. Our data revealed axonal pTDP-43–positive accumulations in approximately one-half of bundles in muscle tissues of patients with autopsy-confirmed SALS, and more than one-half for patients who were ultimately diagnosed with SALS after muscle biopsy. In addition, muscle tissues of patients without ALS did not exhibit pTDP-43–positive intramuscular nerve bundles. These findings strongly suggest that axonal pTDP-43 accumulations may be specific for patients with SALS, as was speculated by Altman et al.^15^

It is anticipated that pTDP-43 and Ras GTPase-activating protein-binding protein may serve as targetable proteins for therapeutic development.^15^ Altman et al reported pTDP-43 accumulations in the muscle tissues of patients with both ALS and non-ALS diseases, detected via immunofluorescence.^15^ Although we also observed such axonal pTDP-43 accumulations in patients with ALS in both the postmortem study and the muscle biopsy cohort, we did not detect any for patients with non-ALS diseases through the immunohistochemistry provided by the autoimmunostaining system. We consider that the immunohistochemical approach for detecting nerve bundles is superior to the immunofluorescent analysis in longitudinal sections utilized for diagnostic use in previous reports.^10^ As such, our results suggest that the autoimmunostaining system may establish a condition for distinguishing patients with ALS from those with non-ALS diseases and, when employed in combination with the Gold Coast criteria, enable us to diagnose ALS more easily. We also observed that intramuscular nerve bundles were more frequently preserved in patients with ALS than in patients with other diseases, which might be another specific feature of ALS. However, this speculation needs confirmation by further prospective studies.

### Limitations

This study had several limitations. First, although previous studies have shown that muscular pTDP-43 accumulations frequently appear in the diaphragm and paraspinal muscles,^18^ paraspinal muscles were not evaluated in these cohorts of patients with autopsy-confirmed or biopsied SALS because the muscle biopsies in this study were not performed for diagnosing ALS. Second, this study excluded patients having pathogenic variants in known causative genes of familial ALS. However, such genetic analysis was not performed in about 70% of patients in groups B and C. Third, this was not a multicenter study and thus needs confirmation by other groups. These limitations mean further examinations are needed to evaluate the sensitivity and specificity of our method.

## Conclusions

Results of this dual case-control and cohort study suggest that axonal pTDP-43 accumulations may be characteristic for patients with ALS, and as a result, may be a novel diagnostic biomarker for ALS. Axonal pTDP-43–positive accumulations were detected not only in the nerve bundles of patients with autopsy-confirmed SALS, but also in those of patients with SALS who had only LMN symptoms as clinical manifestations at the time of biopsy. Further prospective study of axonal pTDP-43 accumulations in intramuscular nerve bundles is needed to understand the pathomechanism of LMN dysfunction in ALS and to clarify the utility of this potential diagnostic test for ALS.

## Notes

Supplement. **eFigure 1.** Immunofluorescent Reactivity Against pTDP-43 in Muscle Tissues of Patients in the Postmortem Study **eFigure 2.** Immunofluorescent Reactivity Against TDP-43 in Muscle Tissues of Patients in the Postmortem Study **eFigure 3.** Immunofluorescent Reactivity Against FUS in Muscle Tissues of Patients in the Postmortem Study **eFigure 4.** Immunofluorescent Reactivity Against SQSTM1/p62 in Muscle Tissues of Patients in the Postmortem Study **eFigure 5.** Immunofluorescent Reactivity Against Ub in Muscle Tissues of Patients in the Postmortem Study **eFigure 6.** Comparison of the Percentage of Intramuscular Nerve Bundles With Axonal pTDP-43 Accumulations Among Autopsy-Confirmed Patients **eFigure 7.** Immunofluorescent Reactivity Against pTDP-43 in Muscle Tissues of Patients in the Muscle Biopsy Cohort **eFigure 8.** Percentage of Patients Receiving a Final ALS Diagnosis Among the 3 Groups **eFigure 9.** Percentage of Nerve Bundles Containing Axonal pTDP-43 Accumulations and Clinical Category at Muscle Biopsy for Groups A and B **eTable.** Clinical and Pathological Characteristics of Patients in the Postmortem Study
