# Supplementary material for: TDP-43 Accumulation Within Intramuscular Nerve Bundles of Patients With Amyotrophic Lateral Sclerosis
Source: JAMA Neurol. 2022 May 23:e221113. Online ahead of print. doi: 10.1001/jamaneurol.2022.1113 (PMC9127711; doi:10.1001/jamaneurol.2022.1113)
Supplement: Supplement. — eFigure 1. Immunofluorescent Reactivity Against pTDP-43 in Muscle Tissues of Patients in the Postmortem Study eFigure 2. Immunofluorescent Reactivity Against TDP-43 in Muscle Tissues of Patients in the Postmortem Study eFigure 3. Immunofluorescent Reactivity Against FUS in Muscle Tissues of Patients in the Postmortem Study eFigure 4. Immunofluorescent Reactivity Against SQSTM1/p62 in Muscle Tissues of Patients in the Postmortem Study eFigure 5. Immunofluorescent Reactivity Against Ub in Muscle Tissues of Patients in the Postmortem Study eFigure 6. Comparison of the Percentage of Intramuscular Nerve Bundles With Axonal pTDP-43 Accumulations Among Autopsy-Confirmed Patients eFigure 7. Immunofluorescent Reactivity Against pTDP-43 in Muscle Tissues of Patients in the Muscle Biopsy Cohort eFigure 8. Percentage of Patients Receiving a Final ALS Diagnosis Among the 3 Groups eFigure 9. Percentage of Nerve Bundles Containing Axonal pTDP-43 Accumulations and Clinical Category at Muscle Biopsy for Groups A and B eTable. Clinical and Pathological Characteristics of Patients in the Postmortem Study [file jamaneurol-e221113-s001.pdf]

Supplementary Online Content

Kurashige T, Morino H, Murao T, et al. TDP-43 accumulation within intramuscular nerve bundles of patients with amyotrophic lateral sclerosis. *JAMA Neurol.* Published online May 23, 2022. doi:10.1001/jamaneurol.2022.1113

eFigure 1. Immunofluorescent Reactivity Against pTDP-43 in Muscle Tissues of Patients in the Postmortem Study
eFigure 2. Immunofluorescent Reactivity Against TDP-43 in Muscle Tissues of Patients in the Postmortem Study
eFigure 3. Immunofluorescent Reactivity Against FUS in Muscle Tissues of Patients in the Postmortem Study
eFigure 4. Immunofluorescent Reactivity Against SQSTM1/p62 in Muscle Tissues of Patients in the Postmortem Study
eFigure 5. Immunofluorescent Reactivity Against Ub in Muscle Tissues of Patients in the Postmortem Study
eFigure 6. Comparison of the Percentage of Intramuscular Nerve Bundles With Axonal pTDP-43 Accumulations Among Autopsy-Confirmed Patients
eFigure 7. Immunofluorescent Reactivity Against pTDP-43 in Muscle Tissues of Patients in the Muscle Biopsy Cohort
eFigure 8. Percentage of Patients Receiving a Final ALS Diagnosis Among the 3 Groups
eFigure 9. Percentage of Nerve Bundles Containing Axonal pTDP-43 Accumulations and Clinical Category at Muscle Biopsy for Groups A and B
eTable. Clinical and Pathological Characteristics of Patients in the Postmortem Study

This supplementary material has been provided by the authors to give readers additional information about their work.

© 2022 Kurashige T et al. *JAMA Neurology.*

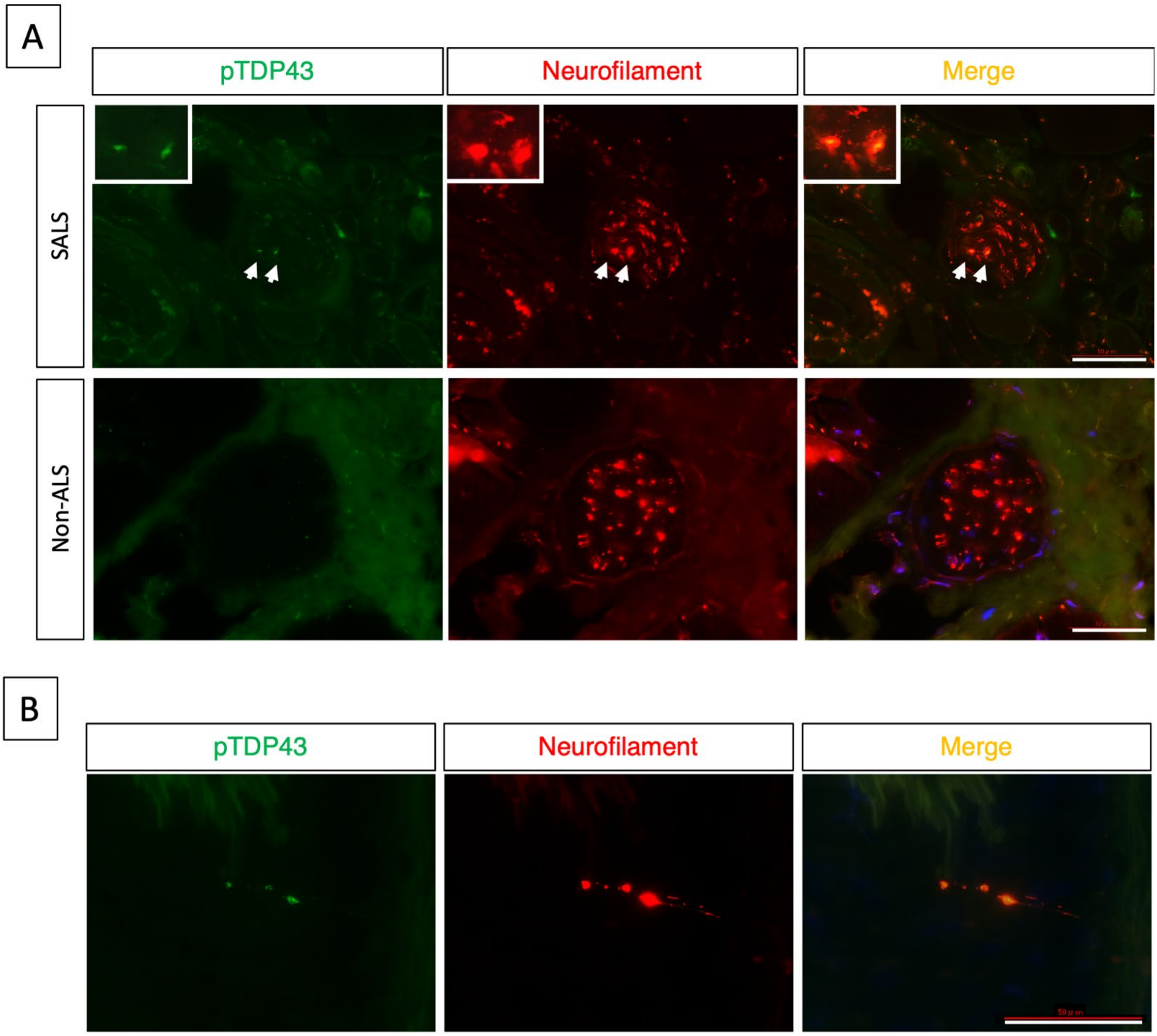

eFigure 1. Immunofluorescent Reactivity Against pTDP-43 in Muscle Tissues of Patients in the Postmortem Study

Immunofluorescent study showed that the muscle tissues of autopsy-confirmed ALS patients presented axonal accumulations stained with the mouse monoclonal antibody against pTDP43 (arrow) in intramuscular nerve bundles (A) and peripheral axons (B).

Scale Bars: 50 μm

© 2022 Kurashige T et al. *JAMA Neurology.*

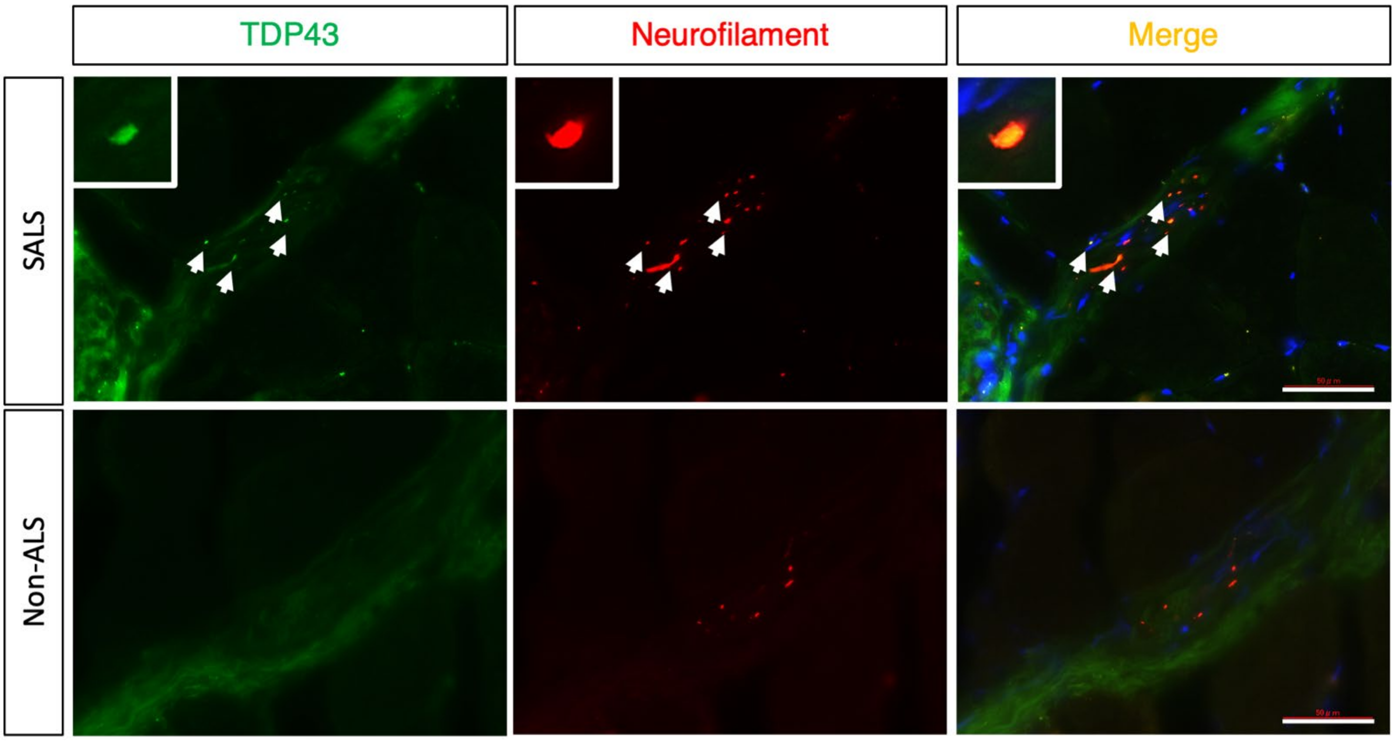

eFigure 2. Immunofluorescent Reactivity Against TDP-43 in Muscle Tissues of Patients in the Postmortem Study

Immunofluorescent study showed that the muscle tissues of autopsy-confirmed ALS patients presented axonal accumulations stained with the rabbit polyclonal antibody against TDP43 (arrow) in intramuscular nerve bundles.

Scale Bars: 50 μm

© 2022 Kurashige T et al. *JAMA Neurology.*

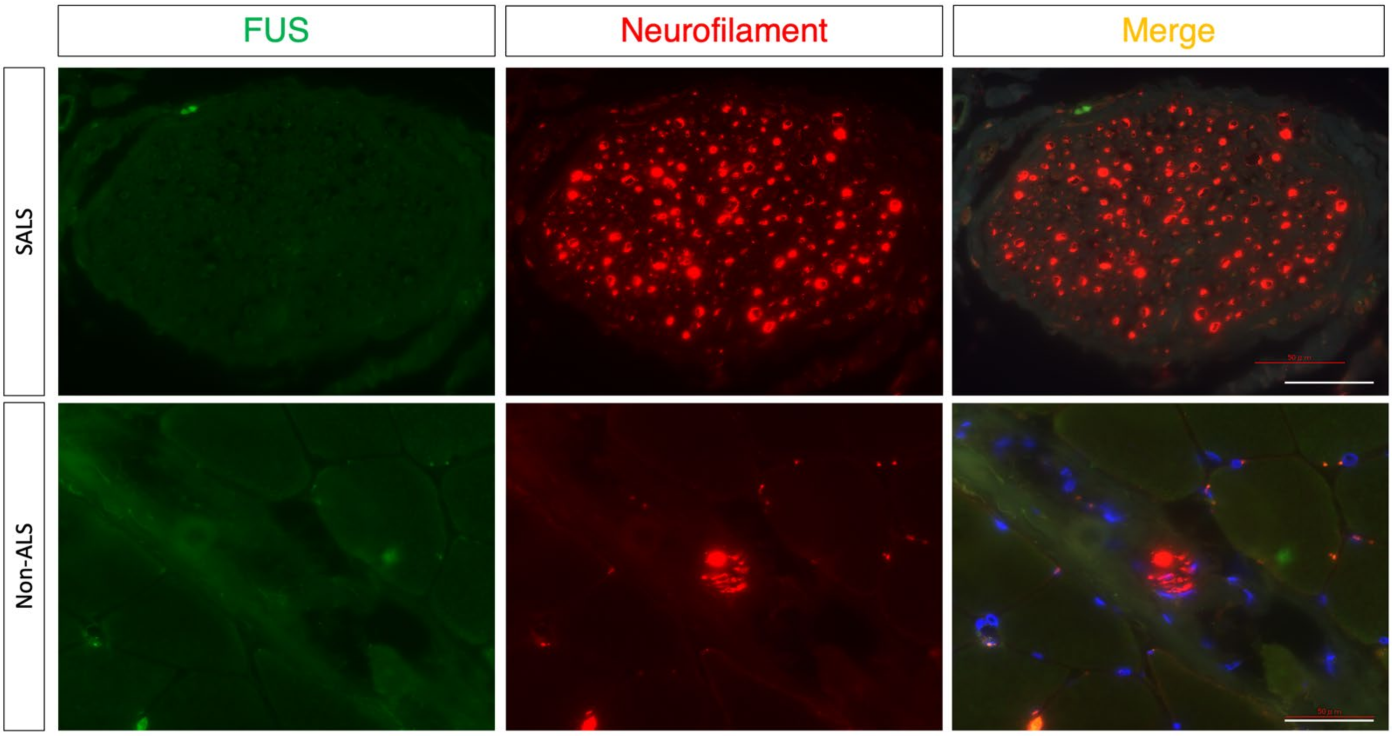

eFigure 3. Immunofluorescent Reactivity Against FUS in Muscle Tissues of Patients in the Postmortem Study

Immunofluorescent study showed that the muscle tissues of autopsy-confirmed patients with SALS and non-ALS diseases did not show any accumulations of FUS.

Scale Bars: 50 µm

© 2022 Kurashige T et al. *JAMA Neurology.*

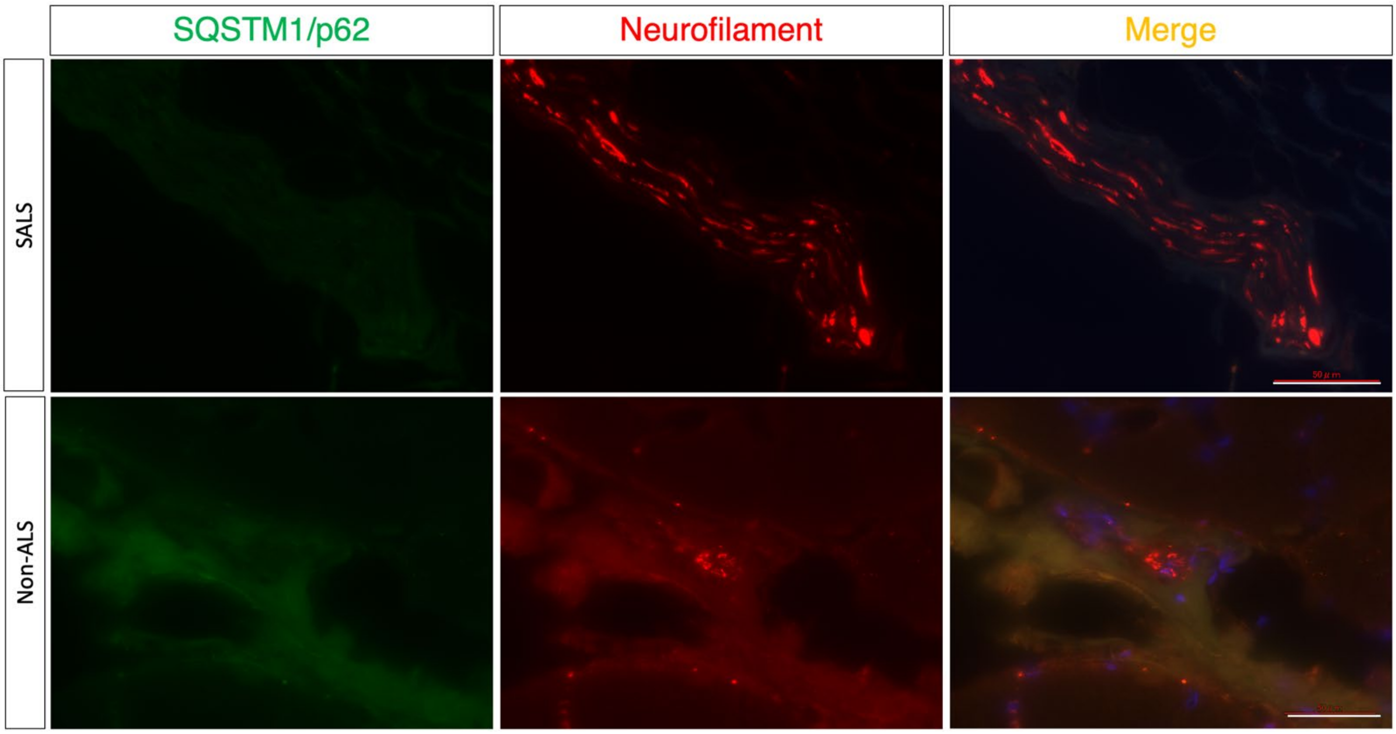

eFigure 4. Immunofluorescent Reactivity Against SQSTM1/p62 in Muscle Tissues of Patients in the Postmortem Study

Immunofluorescent study showed that the muscle tissues of autopsy-confirmed patients with SALS and non-ALS diseases did not show any accumulations of SQSTM1/p62.

Scale Bars: 50 µm

© 2022 Kurashige T et al. *JAMA Neurology.*

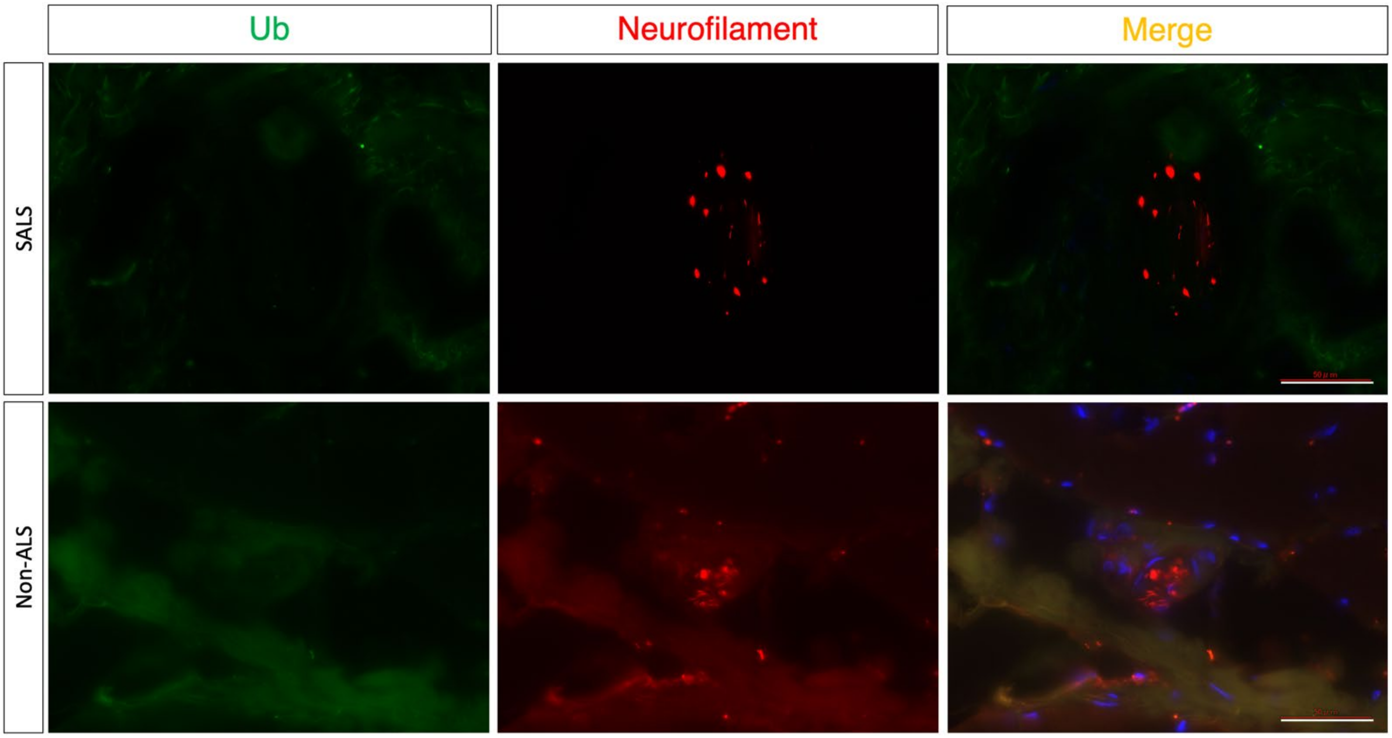

eFigure 5. Immunofluorescent Reactivity Against Ub in Muscle Tissues of Patients in the Postmortem Study

Immunofluorescent study showed that the muscle tissues of autopsy-confirmed patients with SALS and non-ALS diseases did not show any accumulations of Ub.

Scale Bars: 50 μm

© 2022 Kurashige T et al. *JAMA Neurology.*

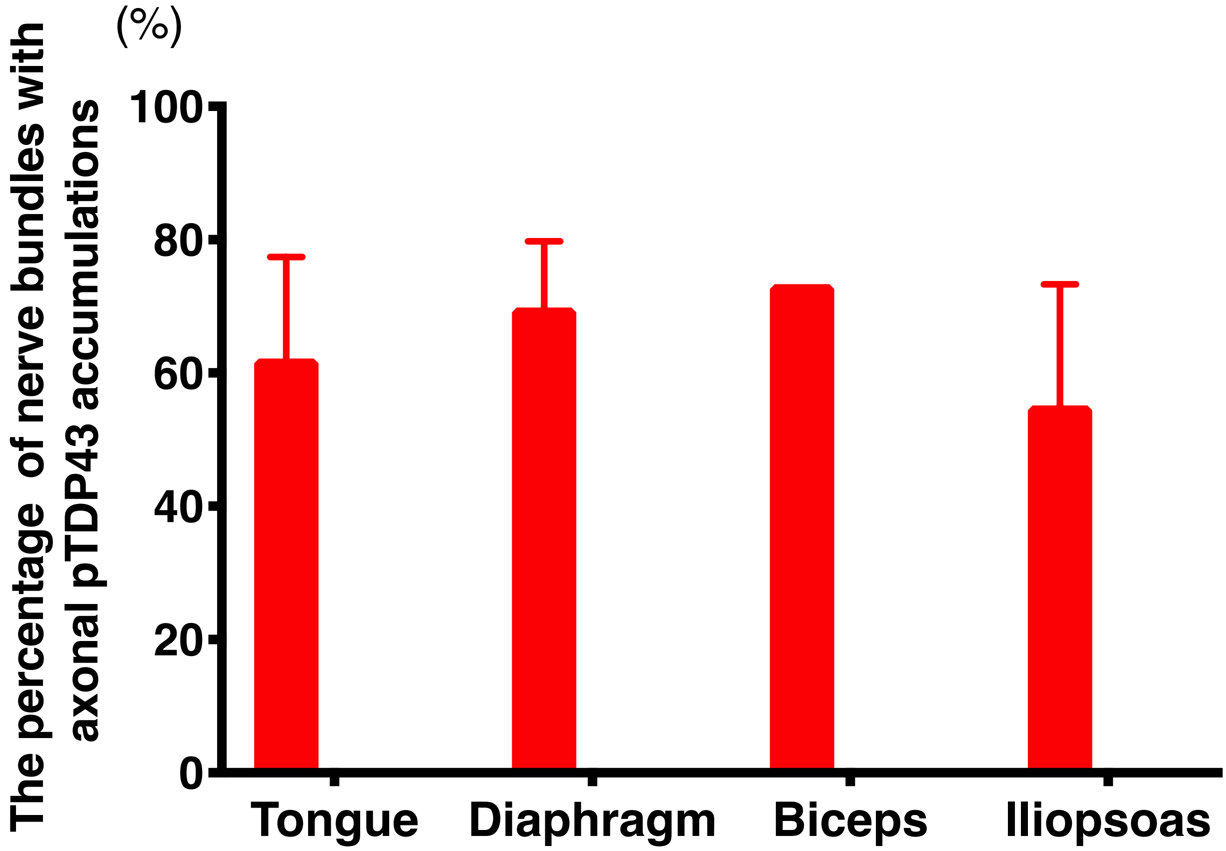

ALS	n = 10	n = 10	n = 1	n = 10
Non-ALS	n = 4	n = 12	n = 3	n = 12

eFigure 6. Comparison of the Percentage of Intramuscular Nerve Bundles With Axonal pTDP-43 Accumulations Among Autopsy-Confirmed Patients

Comparison of the percentage of intramuscular nerve bundles with axonal pTDP43 accumulations among autopsy-confirmed patients. No significant difference was observed among the four types of muscles examined in this study ($P = 0.149$).

© 2022 Kurashige T et al. *JAMA Neurology.*

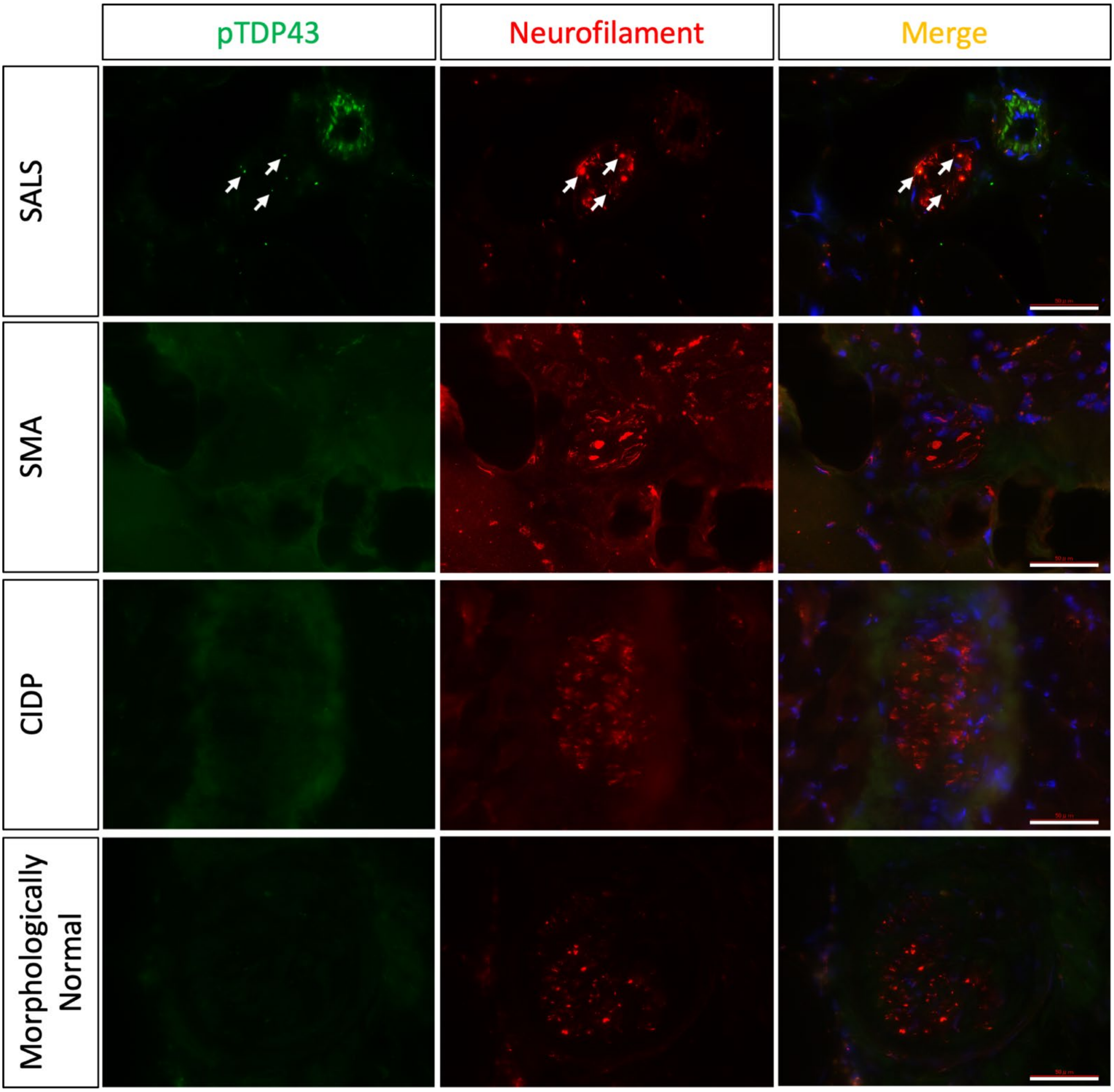

eFigure 7. Immunofluorescent Reactivity Against pTDP-43 in Muscle Tissues of Patients in the Muscle Biopsy Cohort

Immunofluorescent study for pTDP43 in muscle biopsy specimens. pTDP43-positive accumulations in intramuscular nerve bundles were colocalized with axons stained by an anti-neurofilament antibody (arrow) in patients whose clinical symptoms were categorized in probable or definite ALS and diagnosed as SALS finally.

Scale Bars: 50 μm

© 2022 Kurashige T et al. *JAMA Neurology.*

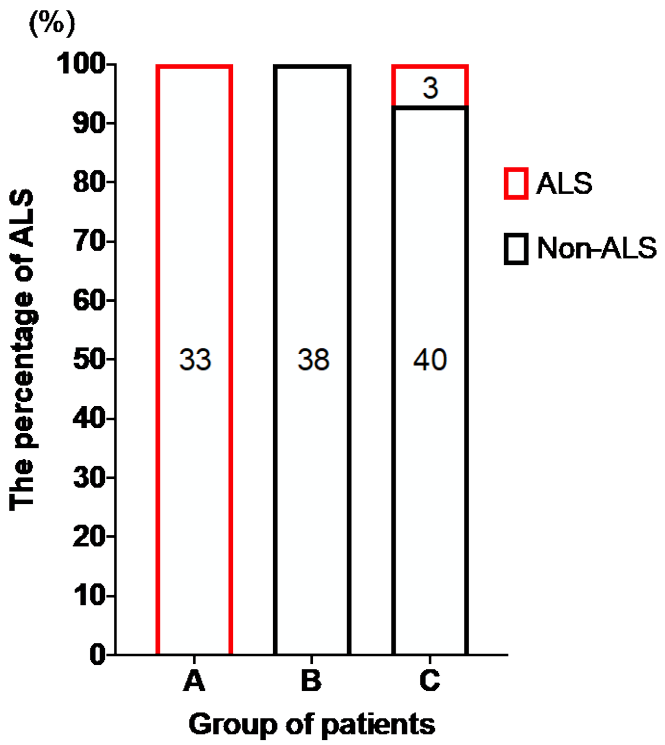

eFigure 8. Percentage of Patients Receiving a Final ALS Diagnosis Among the 3 Groups

Percentage of patients receiving a final ALS diagnosis among the three groups.

© 2022 Kurashige T et al. *JAMA Neurology.*

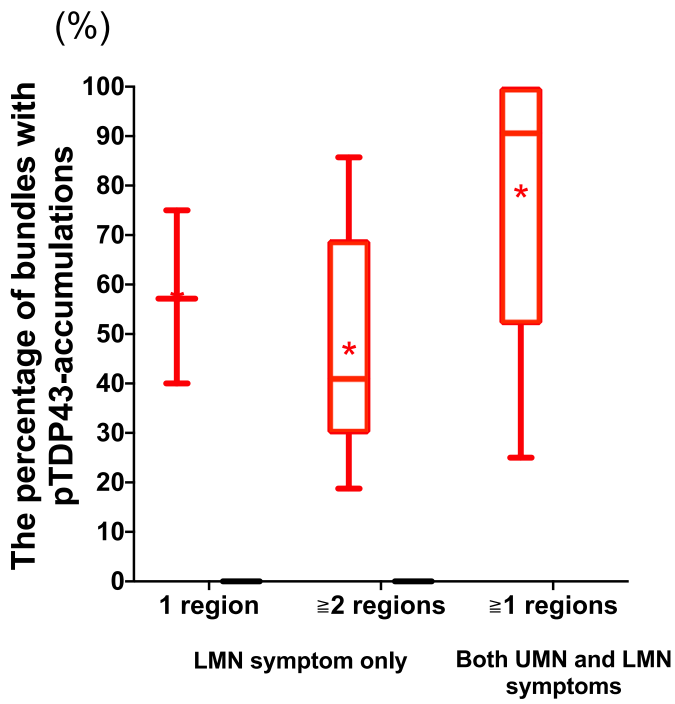

ALS	n = 3	n = 6	n = 24
Non-ALS	n = 16	n = 22	n = 0

eFigure 9. Percentage of Nerve Bundles Containing Axonal pTDP-43 Accumulations and Clinical Category at Muscle Biopsy for Groups A and B

Percentage of nerve bundles containing axonal pTDP43 accumulations and clinical category at muscle biopsy for Groups A and B. Among ALS patients, the percentage of nerve bundles containing axonal pTDP43-positive accumulations significantly increased with UMN symptoms at the same body region of LMN symptoms at muscle biopsy ($p = 0.0050$). Non-ALS patients did not show any pTDP43-poisitive accumulations. Asterisks indicate mean values; bars inside boxes indicate median values.

© 2022 Kurashige T et al. *JAMA Neurology.*

eTable. Clinical and Pathological Characteristics of Patients in the Postmortem Study

Case	Postmortem diagnosis	Clinical diagnosis of ALS subtype	Sex	Age at death (y.o.)	Disease Duration (mo)	pTDP43 positive nerve bundles / all bundles (%)			
						Tongue	Diaphragm	Biceps	Iliopsoas
SALS1	ALS with TDP pathology	bulbar	M	mid-60s	6	26/41 (63.4)	43/68 (63.2)	n.e.	5/ 8 (62.5)
SALS2	ALS with TDP pathology	frail arm	M	late 80s	6	33/56 (58.9)	51/70 (72.8)	n.e.	8/14 (57.1)
SALS3	ALS with TDP pathology	frail arm	M	late 80s	7	18/42 (42.9)	60/71 (84.5)	11/15 (73.3)	6/14 (42.9)
SALS4	ALS with TDP pathology	lower	M	early 70s	14	39/56 (69.6)	37/51 (72.5)	n.e.	11/17 (64.7)
SALS5	ALS with TDP pathology	frail arm	M	early 60s	15	27/57 (47.4)	34/66 (51.5)	n.e.	7/19 (36.8)
SALS6	ALS with TDP pathology	bulbar	M	early 80s	16	41/51 (80.4)	45/55 (81.8)	n.e.	10/13 (76.9)
SALS7	ALS with TDP pathology	bulbar	F	mid-70s	24	32/37 (86.5)	45/67 (67.2)	n.e.	6/18 (33.3)
SALS8	ALS with TDP pathology	bulbar	M	early 70s	32	36/51 (70.6)	34/44 (77.3)	n.e.	5/11 (45.5)
SALS9	ALS with TDP pathology	lower	F	early 80s	110	5/12 (41.7)	7/11 (63.6)	n.e.	8/ 9 (88.9)
SALS10	ALS with TDP pathology	bulbar	M	mid-70s	19	23/38 (60.5)	14/22 (63.6)	n.e.	3/ 7 (42.9)
Cont1	Paraneoplastic syndrome		M	mid-50s		0/62 (0.0)	0/20 (0.0)	0/28 (0.00)	0/13 (0.0)
Cont2	normal (AMI)		M	mid-60s		n.e.	0/23 (0.0)	n.e.	0/14 (0.0)
Cont3	Parkinson disease		M	early 80s		0/41 (0.0)	0/36 (0.0)	0/36 (0.0)	0/12 (0.0)
Cont4	CNS lymphoma		M	mid-80s		0/36 (0.0)	0/18 (0.0)	n.e.	0/16 (0.0)
Cont5	SAH		M	late 40s		n.e.	0/16 (0.0)	n.e.	0/19 (0.0)
Cont6	Parkinson disease		M	early 60s		0/47 (0.0)	0/28 (0.0)	n.e.	0/13 (0.0)
Cont7	AQP4-positive NMOSD		M	early 60s		n.e.	0/33 (0.0)	n.e.	0/11 (0.0)
Cont8	Subdural hematoma		M	early 80s		n.e.	0/34 (0.0)	n.e.	0/14 (0.0)

© 2022 Kurashige T et al. *JAMA Neurology.*

Cont9	Cerebral infarct		M	mid-70s		n.e.	0/25 (0.0)	n.e.	0/ 9 (0.0)
Cont10	Cerebral infarct		M	late 70s		n.e.	0/22 (0.0)	n.e.	0/10 (0.0)
Cont11	CBD		M	mid-70s		n.e.	0/28 (0.0)	n.e.	0/16 (0.0)
Cont12	PSP		M	early 80s		n.e.	0/51 (0.0)	n.e.	0/21 (0.0)

Abbreviations: ALS, amyotrophic lateral sclerosis; SALS, sporadic ALS; Cont, control; TDP, TAR DNA-binding protein (TARDBP) encoding TAR DNA-binding protein 43; pTDP43, phosphorylated TDP43; AMI, acute myocardial infarction; CNS, central nervous system; SAH, subarachnoidal hemorrhage; AQP4, aquaporin-4; NMOSD, neuromyelitis optica spectrum disorder; CBD, corticobasal degeneration; PSP, progressive supranuclear pulsy

© 2022 Kurashige T et al. *JAMA Neurology.*
